# Saffron’s protective role against atherosclerosis-induced cataract progression in New Zealand white rabbits with phytochemical analysis of saffron’s extract

**DOI:** 10.1371/journal.pone.0315178

**Published:** 2025-01-14

**Authors:** Yasmin Mohd Zainal Abidin Shukri, Iman Nabilah Abd Rahim, Nurul Alimah Abdul Nasir, Che Puteh Osman, Noor Alicezah Mohd Kasim

**Affiliations:** 1 Laboratory and Forensic Medicine (I-PPerForM), Institute of Pathology, Universiti Teknologi MARA, Sungai Buloh, Selangor, Malaysia; 2 Faculty of Medicine, Department of Pathology, Universiti Teknologi MARA, Sungai Buloh, Selangor, Malaysia; 3 Faculty of Medicine, Department of Pharmacology, Universiti Teknologi MARA, Sungai Buloh, Selangor, Malaysia; 4 Atta-ur-Rahman Institute for Natural Product Discovery (AuRIns), Universiti Teknologi MARA, Cawangan Selangor, Kampus Puncak Alam, Bandar Puncak Alam, Selangor, Malaysia; 5 Faculty of Applied Sciences, Universiti Teknologi MARA, Shah Alam, Selangor, Malaysia; The University of Tennessee Health Science, UNITED STATES OF AMERICA

## Abstract

Cataracts are significant causes of blindness, closely linked to prolonged hypercholesterolemia. While saffron has the potential for eye health, its effects on lens lesions remain understudied. This study aimed to investigate the effect of saffron on the lens changes in atherosclerotic-induced New Zealand white rabbits (NZWR). Thirty-five NZWRs were subjected to four to eight weeks of high-cholesterol diet to induce atherosclerosis, resulting in cataractous lens changes. The rabbits were categorised randomly into three groups: normal diet group, pre-treated group and treated group. The pre-treated group was divided into early atherosclerosis(HC4) and established atherosclerosis (HC8). The saffron-treated group was fed with the HCD diet followed by saffron treatment of 50mg/kg/day (TG450, TG840) and 100mg/kg/day (TG4100, TG8100) of saffron ethanolic extract (SEE) respectively. The normal diet group was given a normal diet over the 8 weeks. After completing the 16-week experimental protocol, the NZWR were euthanized, and their lenses were extracted for histopathological evaluation. The pre-treated group exhibited cataractous lens changes of grade 2, characterized by increased homogenisation, swollen lens fibers, and intracellular vacuolisation. Interestingly, these cataract changes showed a positive trend from grade 2 to grade 1 post-treatment with SEE. In the saffron-treated group, vacuoles and pinkish homogenised areas were reduced. Additionally, a uniform layer of anterior epithelium and decreased non-swollen lens fibers indicated significant cataract lesion improvement. The normal diet group displayed minimal to zero cataractous changes (Grade 0). HPLC analysis demonstrated the presence of crocin, crocetin, and picocrocin in the saffron ethanolic extract, with peak absorptions at 440nm (12.817min), 440nm (1.620min), and 254nm (6.553min) respectively. The phytochemical screening of saffron ethanolic extract was conducted and showed the presence of phytochemical compounds including saponins, flavonoids, tannins, and steroids. The positive effects on lenses in the TG groups could be due to crocin and crocetin, bioactive components of saffron, and its phytochemical compounds. This study highlights saffron’s potential in managing cataract-induced conditions, emphasizing the importance of further research for its full therapeutic potential in cataract management.

## Introduction

According to the World Health Organization (WHO), at least 2.2 billion people are suffering from vision impairment. Among them, cataract was the leading cause of blindness, accounting for 94 million people. The number of cataract cases increased in Malaysia, which has a detrimental effect on the growth of the national economy and raises healthcare expenses [1,2]. Moreover, the inability to carry out daily tasks is greatly affected by vision loss, resulting in both functional and psychological distress [1]. A cataract is defined as the clouding or opacification of the clear lens of the eye or its capsule (the transparent membrane surrounding the lens), obstructing light flow to the retina and lens [3]. Over time, the illness gradually worsens, extending and becoming more defined. Additionally, the disease results in structural and functional alterations to the structure of the eye, which eventually cause blindness and symptoms like glare and halos [3,4]. Past research reported that 86% of the major causes of blindness could have been prevented and 58% of them could be treated [1]. Lens extraction surgery is considered to be the most common ocular procedure and most effective treatment for cataracts [1]. Having cataract surgery necessitates higher costs, and highly skilled surgeons, in both local and private hospitals.

Hypercholesterolemia or hyperlipidemia is known to be associated in the development of cataract [5,6]. Several research studies emphasised the correlation between elevated levels of cholesterol and increased susceptibility to developing cataracts [5,7]. The underlying molecular pathways implicated in this correlation may encompass oxidative stress and lipid peroxidation, processes capable of detrimentally impacting lens proteins and fibers, consequently leading to opacification [8,9]. Elevated cholesterol levels in hypercholesterolemic mice showed increase oxidation of phospholipids, promoting inflammatory reactions [10]. When LDL becomes oxidized (forming oxidized LDL, or oxLDL), it contributes to plaque buildup in arteries and also produces oxidized phospholipids (OxPL), which intensify inflammation and cellular damage [10,11]. Numerous studies have highlighted the key role of oxidative stress in cataract formation, including its damage on lens epithelial cells (LECs) [8,11]. Additionally, animal studies have demonstrated that a high-cholesterol diet triggers oxidative stress by activating inflammatory and proliferative pathways, further accelerating the process [12,13]. A study demonstrated that hypercholesterolemia, particularly in individuals with type 2 diabetes, is associated with a significantly higher risk of cataract formation compared to those with clear lenses [14]. Moreover, a statistically significant correlation has been observed between hypercholesterolemia and the presence of cortical cataracts [15]. Cataract patients were found to exhibit significantly higher serum cholesterol levels compared to healthy controls, further reinforcing the association between hypercholesterolemia and cataractogenesis [15,16]. Histological examinations reveal an increase in lipid concentration at the onset of cataract development, resulting in alterations in intramembrane lipids that are intricately associated with the pathogenesis of cataracts. These results emphasise the crucial necessity of efficiently managing hyperlipidemia within a comprehensive framework aimed at reducing the risk of cataracts, particularly within demographics showing an increased prevalence of metabolic disorders [17,18].

The relationship between coronary heart disease (CHD) and cataracts has been investigated in numerous research studies [19,20]. Various studies have reported that individuals with cataracts who receive surgical treatment show a heightened occurrence of cardiovascular conditions and risk factors, such as diabetes, hypertension, and hyperlipidemia [20]. Additionally, the oxidative degradation of proteins in the lens, which is also a key factor in the development of age-related cataracts, could also serve as an indicator of an increased risk of future CHD [21]. Furthermore, genetic investigations have demonstrated notable connections between congenital cataracts and congenital heart anomalies [21].

Saffron is the dried stigmas of the stemless herb *Crocus sativus* L. flower, which is a member of the Iridaceae family [22]. Saffron is an extravagant spice that’s widely recognised for its use in traditional cuisines as a natural coloring and flavoring [23,24]. At the same time, saffron has healing properties as it is well known that it has been used traditionally to treat a variety of illnesses and wounds [25]. The primary components of saffron, namely crocin, crocetin, picocrocin, and safranal, are naturally occurring carotenoid compounds that have been shown to exhibit a variety of characteristics, including anti-inflammatory, anti-oxidative, and neuroprotective effects [26]. These generated a lot of interest in studying the health benefits of saffron and its mechanism through research [27]. Past studies have demonstrated that saffron supplementation has significantly improved ocular diseases, such as age-related macular degeneration, glaucoma, and diabetic maculopathy [28,29]. Nevertheless, the functional role of saffron in the progression of cataracts has not been extensively studied. Despite increasing literature reporting the potential benefits of saffron intervention in eye conditions, there is a significant scarcity of data concerning the effects of saffron on atherosclerotic-induced cataracts in rabbits.

Therefore, this study aimed to investigate the effect of saffron on the lens changes in atherosclerotic-induced New Zealand white rabbits(NZWR). Additionally, this study aimed to conduct qualitative and quantitative phytochemical analysis of saffron ethanolic extract using High Performance Liquid Chromatography (HPLC), to quantify bioactive compounds includes crocin, crocetin and picocrocin.

## Materials and methods

### Saffron extract preparation

Saffron stigma was purchased from World Care Groups Sdn Bhd and was verified by Herbarium Universiti Kebangsaan Malaysia (UKMB) (voucher number: ID006/2021). A quantity of 10 g of saffron stigma were blended using an electric blender and were soaked in 500 mL of ethanol: water (80:20, v/v) and stirred using a magnetic stirrer for 3 days under room temperature. The mixture was filtered and further purified and concentrated using a rotary evaporator at 50°C with a mixing speed of 120 rpm. The resulting crude extract was kept at -80°C overnight and was then lyophilized using a freeze-drying process. The saffron ethanolic extract (SEE) produced was kept at 4°C before use.

### HPLC analysis

**Fig 1:**
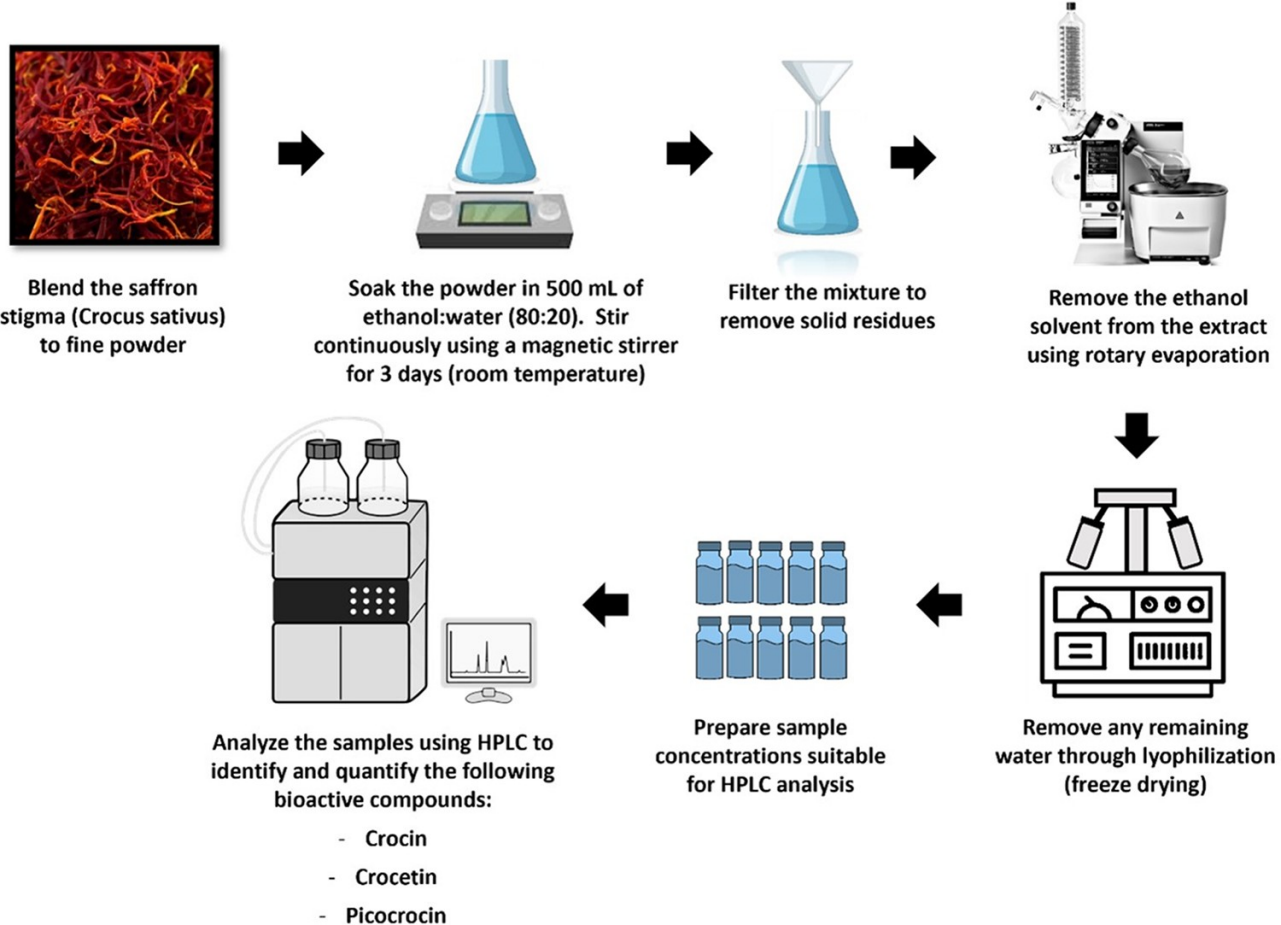
Experimental outline for saffron extraction and HPLC analysis.

Stock solutions of crocin, picrocrocin, and crocetin at a concentration of 1,000 ppm were prepared, respectively. A series of dilutions of the solutions with a concentration range of 4 ppm to 1000 ppm were prepared for the calibration plot. The samples were analysed using an HPLC system (WATERS 2535 quaternary gradient pump, WATERS 2707 autosampler, and WATERS 2998 PDA) and a Phenomenex Luna C18 HPLC column (5 μm, 250 mm x 4.6 mm) with a gradient system consisting of 2 types of solvents; A (0.1% formic acid in water) and B (acetonitrile). The flow rate was set at 1 mL/min with a sample volume of 10 μL. Retention time data and UV spectra for clear and distinct peaks were analysed and recorded. The analyses were performed in triplicate. The outline for the extraction and HPLC analysis was presented in Fig 1.

### Qualitative phytochemical tests

#### Alkaloids test

The sample was macerated in chloroform followed by the addition of ammoniacal chloroform. The mixture was then treated with 10%sulphuric acid and tested with Mayer’s reagent. The formation of white precipitates indicates the presence of alkaloids.

#### Saponin test

The methanol extract of the sample was mixed with distilled water in a test tube. The formation of stable froth for at least 15 minutes indicates the presence of saponins.

#### Flavonoids test

The chloroform extract of the sample was dissolved in ether and shaken in a 10% ammonia solution. The formation of yellow colour in the ammonia layer indicates the presence of flavonoids.

#### Terpenes test

The chloroform extract was tested using Liebermann-Buchard reagent. The formation of reddish colour indicated the presence of triterpenes and greenish colour for steroids.

#### Steroids test

Chloroform extract was tested using Liebermann-Buchard reagent. Formation of greenish colour indicated the presence of triterpenes and greenish colour for steroids.

#### Tannins test

Ethanolic extract was mixed with 1% ferric chloride solution. The formation of a blue-black colour indicated the presence of hydrolysable tannins, while the brownish-green colour indicated the presence of condensed tannins.

#### Animal experimental protocol

**Fig 2:**
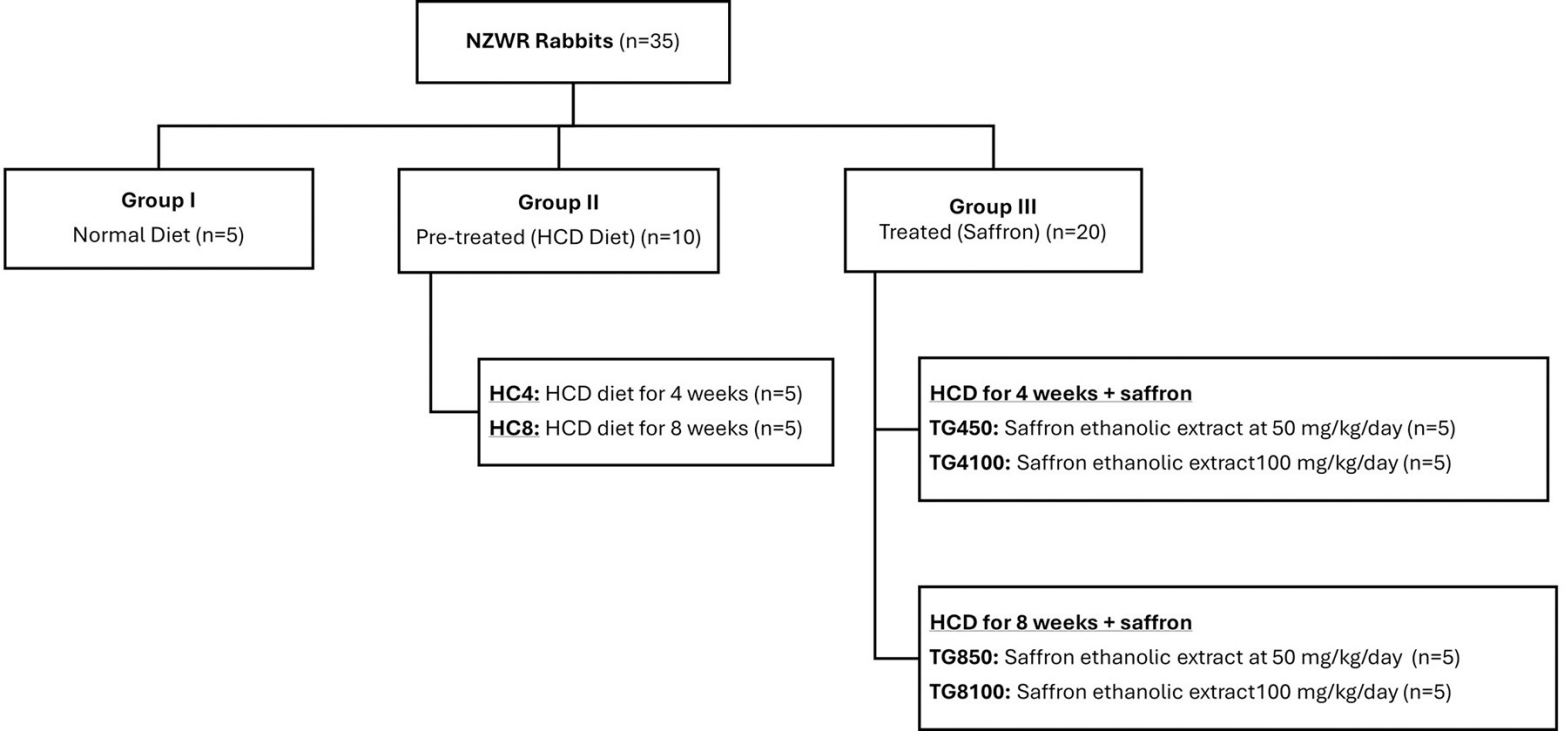
Animal experimental protocol: Rabbit grouping.

Thirty-five male New Zealand white rabbits (NZWR), each weighing between 2–2.2 kg, were purchased from A Sapphire Enterprise, Malaysia. These rabbits were subjected to a high-cholesterol diet (HCD) to induce atherosclerosis and were divided into three main groups which were normal diet, pre-treated, and treatment group. ND received a normal diet (n = 5); the HC4 group received the HCD for 4 weeks to induce early atherosclerosis (n = 5) while the HC8 group received the HCD for 8 weeks to induce established atherosclerosis (n = 5) [30]. The treatment group were further divided into two subgroups. The first treatment group consisted of rabbits that received HCD for 4 weeks to induce early atherosclerosis, followed by an oral administration of saffron ethanolic extract (SEE) at either 50 mg/kg/day (TG450, n = 5) or 100 mg/kg/day (TG4100, n = 5). The second treatment group consisted of rabbits that received the HCD for 8 weeks to induce established atherosclerosis, followed by an oral administration of SEE at 50 mg/kg/day (TG850), n = 5) or 100 mg/kg/day (TG8100, n = 5). The grouping of rabbits was summarized in Fig 2. At the end of the experiment, NZWR rabbits were euthanized humanely with an intravenous dose of sodium pentobarbital (100 mg/kg) to ensure a quick and painless process. Immediately following euthanasia, the cessation of heartbeat and respiratory movement was verified before proceeding with eye extraction, where only one lens per rabbit were extracted for histopathological evaluation. These measures were implemented to minimise any potential suffering. The carcasses were then disposed of following institutional protocols and environmental regulations, ensuring proper biohazard management. The sample size was calculated in alignment with study design recommendations [31]. The study’s duration and sample size were sufficient to establish a hypercholesterolemic state, facilitating observations of early-stage atherosclerosis after a 4-week induction and more advanced progression following an 8-week induction [30]. The experimental protocol of the study was approved by the Universiti Teknologi MARA Committee on Animal Research & Ethics (UiTM CARE) and conducted in compliance with their regulations. The ethical approval number for this study was UiTM CARE: 326/2020.

#### Histopathological analysis

NZWR-extracted lenseswere enucleated and fixed in 10% formalin. The eyes were then dissected sagittally and placed in tissue cassettes for tissue processing using an automated tissue processor MICROM STP120, Thermo Scientific™ (Waltham,MA, USA) over 24 hours. The processed tissues were embedded in paraffin using Tissue-Tek® TEC™ (Torrance, CA, USA) to form paraffin-embedded tissue blocks. These blocks were sectioned into 8–10 micron slices with a rotary microtome (Leica Biosystems Nussloch GmbH, Germany) and mounted on PolysineTM adhesion microscope slides (Epredia Netherlands, Netherlands). The slides were prepared for histopathological and immunohistochemical examination by baking 60°C for 40 minutes followed by deparaffinization in xylene and rehydration through a series of decreasing ethanol concentrations. A standard procedure for Hematoxylin and Eosin (H&E) staining was performed using Tissue-Tek® hematoxylin and eosin (H&E) Staining Kit, Sakura (Torrance,CA, USA). The slides then were examined and imaged using a fluorescent light microscope. The histopathological changes were evaluated using the gradation method (Table 1) [32] by three blinded and independent qualified researchers.

**Table 1:** Grading of cataractous changes adapted from Agarwal et al. (2013) [32].

Gradation	Description
Grade 0	Presence of anterior epithelium with lens fibers
Grade 1	Presence of anterior epithelium, lens fibers and vacuoles
Grade 2	Presence of anterior epithelium, lens fibers, vacuoles and homogenized area
Grade 3	Absence of anterior epithelium, presence of lens fibers, vacuoles and homogenized area
Grade 4	Presence of lens fibers and homogenized area only

## Results

### HPLC analysis

**Fig 3:**
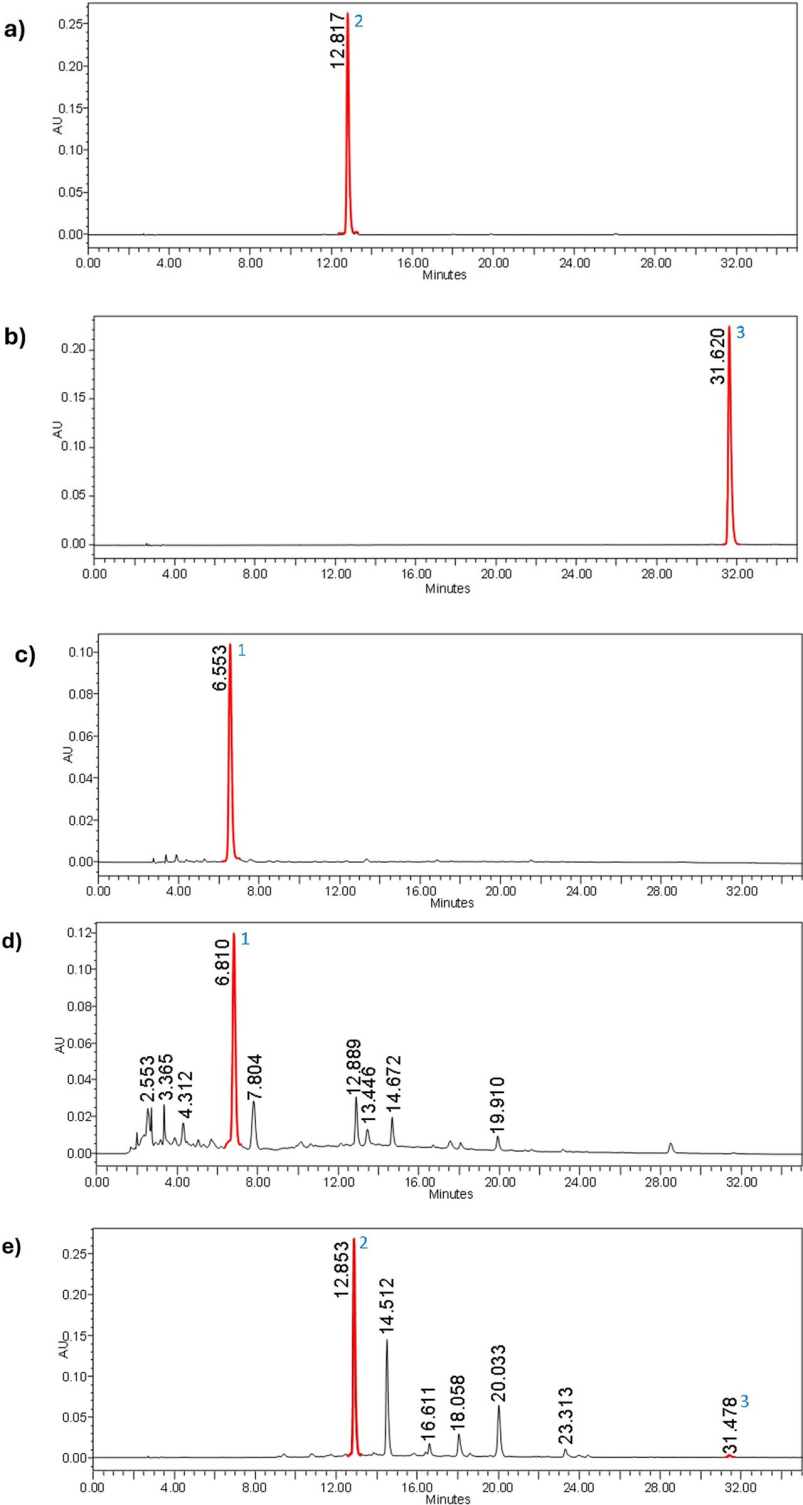
HPLC fingerprint chromatograms of crocin (a), crocetin (b), picocrocin (c). HPLC chromatogram of saffron ethanolic extract (SEE) at 440 nm detection which shows crocin and crocetin at 12.853min and 31.478 respectively. (d) and 254nm detection which shows picocrocin at 6.810min (e).

**Fig 4:**
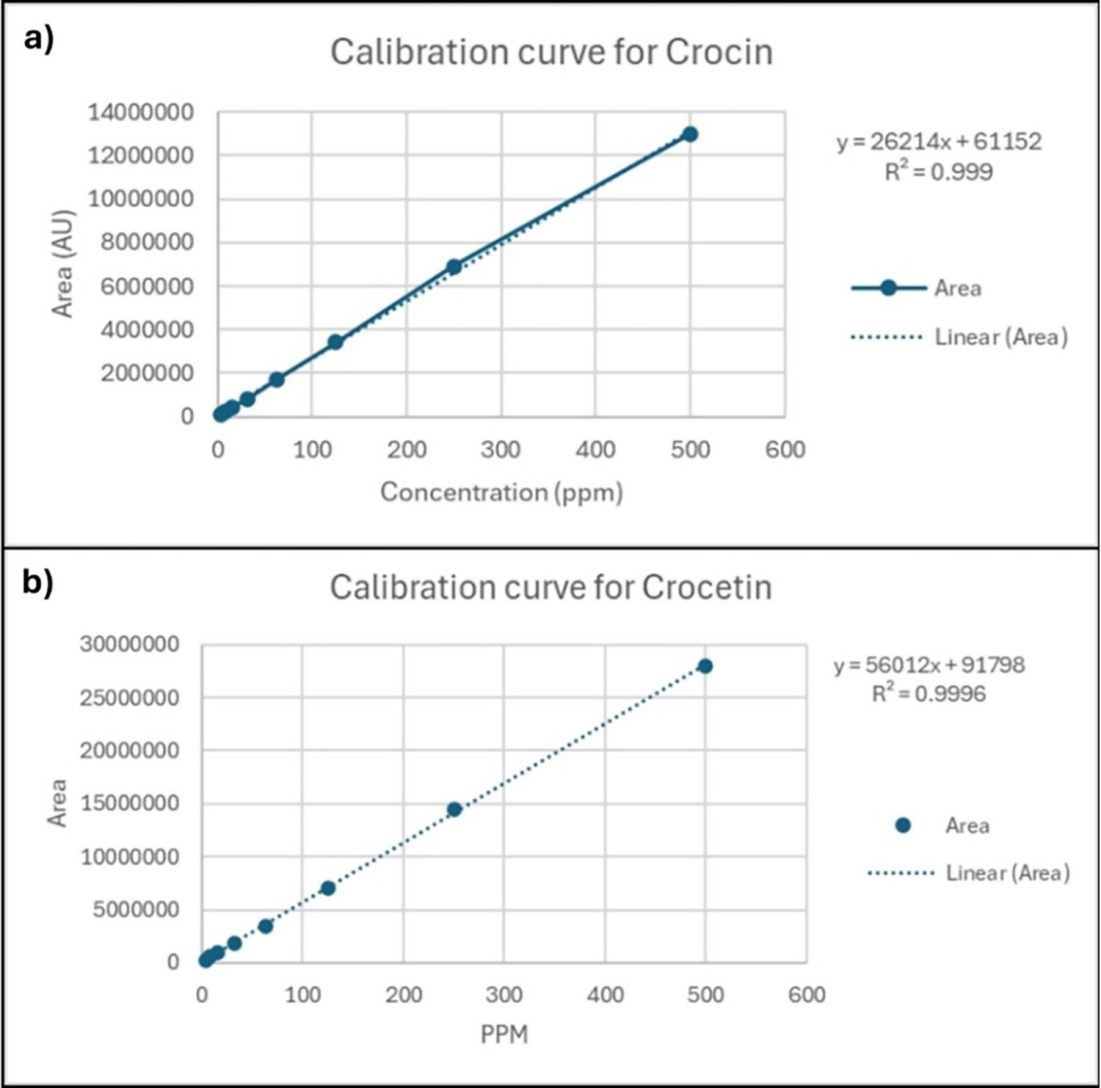
Calibration curve for crocin (a) crocetin (b).

The HPLC analysis of Crocus sativus (saffron) was performed using a gradient method based on the protocol established by García-Rodríguez et al. (2014) to identify the key constituents: crocin, crocetin, and picocrocin. Signals were recorded at 440 nm for crocin and crocetin, and at 254 nm for picocrocin, ensuring precise identification of these compounds. The saffron ethanolic extract was analyzed in comparison to the standards. Retention times (tR) for all detected standards and compounds in the saffron ethanolic extract are provided in Table 2. The close correlation of these retention times with those of the standards strongly confirms the identity of the respective compounds within the extract. Fig 3 illustrates chromatograms obtained from the HPLC analysis, showcasing the distinct peaks corresponding to crocin, crocetin, and picocrocin. Furthermore, calibration curves were constructed to quantify the compounds accurately. By plotting the peak areas against known concentrations of each compound, we achieved robust calibration curves. Fig 4 presents these calibration curves for crocin and crocetin, demonstrating a linear relationship and validating the method’s quantitative capabilities.

**Table 2:** Retention time (t_R_) of crocin, crocetin and picocrocin.

Sample	Crocin	Crocetin	Picocrocin
t_R_ standard (min)	12.82	31.62	6.55
t_R_ extract (min)	12.85	31.48	6.81

### Phytochemical tests

The results of the phytochemical tests are presented in the Table 3. Phytochemical tests showed that saffron ethanolic extract contained secondary metabolites of steroids, terpenes, flavonoids, alkaloids, saponins and tannins. The presence of phenolics and flavonoids compounds supports the results of previous studies which stated that saffron contain flavonoid compounds. Besides that, it supports the potential use of saffron as a medicinal plant.

**Table 3:** Phytochemical tests of saffron ethanolic extract (SEE).

NO.	PHYTOCHEMICALS	RESULTS	CONCLUSION
1	Alkaloids	-	Alkaloids were not detected
2	Saponins	3+	Saponins were detected
3	Flavonoids	3+	Flavonoids were detected
4	Tannins	2+	Tannins were detected
5	Terpenes	-	Terpenes were not detected
6	Steroids	2+	Steroids were detected

*Indications:—(Absent); + (Present, low); 2+ (Present, moderate); 3+ (Present, strong).

### Histopathology changes

**Fig 5:**
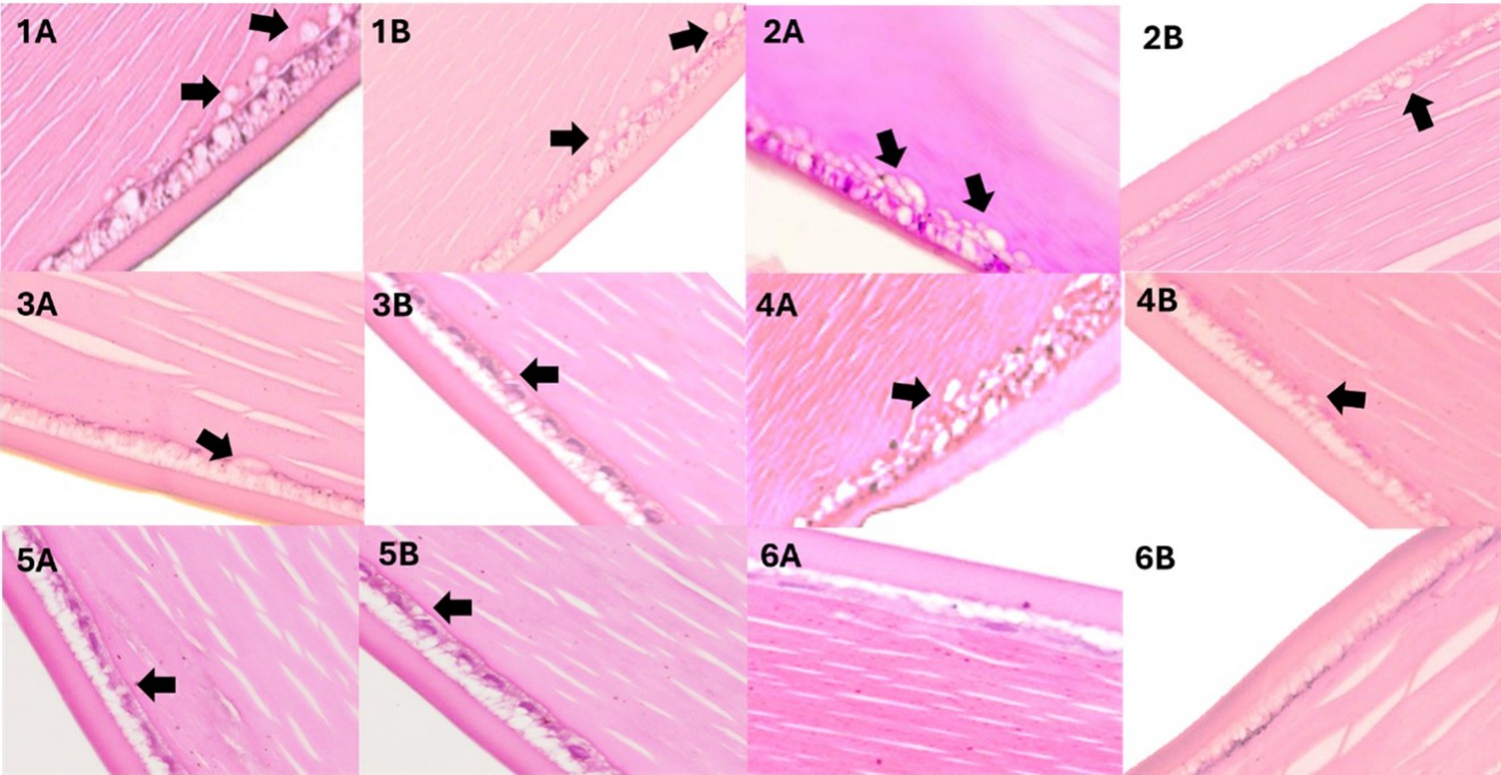
Histopathological changes in pre-treated group, treated group and normal diet group. 1A-1B: Histological structures of treated group (HC4, HC8) respectively. 2A-2B: Histological structures TG450 post treatment. 3A-3B: Histological structures TG4100 post treatment. 4A-4B: Histological structures TG850 post treatment. 5A-5B: Histological structures TG8100 post treatment. 6A-6B: Histological structures of normal diet group (CG). Different degree of vacuolization was observed (arrows).

The results of the cataractous changes were recorded in Table 4 and the histopathological changes in the pre-treated, treated, and normal diet groups were presented in Fig 5. The normal diet (ND) group, which did not receive HCD or saffron supplementation, was maintained on a regular diet for 8 weeks. In this group, 4 out of 5 lenses showed no signs of cataract development. The pre-treated groups, consisting of HC4 and HC8, demonstrated cataract progression in the lenses. In the HC4 group, 3 lenses were graded at 1, while 2 lenses were graded at 2. In the HC8 group, 3 out of 5 lenses exhibited cataractous changes of Grade 2. Both pre-treated groups showed a shift from Grade 0 to higher grades, indicating more advanced cataract formation compared to the ND group. The treated groups were further divided based on the saffron dose (50mg or 100mg). In the TG450, TG4100, and TG8100 groups, 3 lenses were graded at 1 and 2 lenses at Grade 2, respectively. In the TG850 group, 2 lenses showed cataractous changes of Grade 1, and 3 lenses were graded at 2. Therefore, cataract severity was lower in saffron treated group, compared to the HC4 and HC8 groups. tThe saffron-treated groups displayed a similar distribution of cataract grades, suggesting that saffron treatment may have an effect on slowing cataract progression but does not completely prevent it.

**Table 4:** Gradation of lens changes in normal diet, pre-treated and treated groups.

No. of lenses with different degree of cataract changes						
Group	Grade 0	Grade 1	Grade 2	Grade 3	Grade 4	Median (range)
ND (n = 5)	4	1	0	0	0	0
HC4 (n = 5)	0	3	2	0	0	2
HC8 (n = 5)	0	2	3	0	0	2
TG450 (50mg saffron) (n = 5)	0	3	2	0	0	1
TG4100 (100mg saffron) (n = 5)	0	3	2	0	0	1
TG850 (50mg saffron) (n = 5)	0	2	3	0	0	2
TG8100 (100mg saffron) (n = 5)	0	3	2	0	0	1

ND: Normal diet; HC4: High cholesterol-fed diet (4 weeks); HC8: High cholesterol-fed diet (8 weeks); TG450 and TG850: Treatment groups receiving 50 mg/kg/day saffron for 4 and 8 weeks, respectively; TG4100 and TG8100: Treatment groups receiving 100 mg/kg/day saffron for 4 and 8 weeks, respectively.

## Discussion

The therapeutic benefits of natural plant products in related eye diseases are garnering significant attention within the field of ophthalmology and public health [33,34]. Studies have revealed that natural compounds display powerful anti-inflammatory effects, which are crucial in easing inflammation related to cataract progression [35]. Our study results revealed the potential therapeutic advantages of saffron in cataract treatment, showing enhancements in lens modifications within the NZWR-treated group.

The ethanolic saffron extract was optimised using High Peformance Liquid Chronatography. In the present study, High-Performance Liquid Chromatography (HPLC) was employed to investigate the bioactive components in saffron, with a focus on how different compounds separate and are identified. To ensure the purity and authenticity of saffron ethanolic extract, the primary components examined are crocin, crocetin, and picrocrocin [36,37]. Our study confirmed the presence of crocin, crocetin, and picrocrocin in the saffron ethanolic extract used for the intervention group. The peak identifications aligned closely with the retention times of the standard compound where crocin and crocetin were detected at 440 nm, and picrocrocin at 254 nm. These findings are in line with past studies which used similar detection wavelengths for these components [37,38]. Additionally, previous study showed distinct peaks for these compounds in their HPLC analyses, which correlate well with our results [39]. The close alignment of retention times between the standards and the saffron extract indicates the method’s reliability in identifying and quantifying these compounds with minimal interference[40]. From the regression equations and correlation coefficients of the standards, the method demonstrated high linearity. The steep slopes of the calibration curves indicate the sensitivity of the method, while the high R^2^ values reflect its accuracy and dependability across the observed concentration ranges. Our findings are consistent with past studies that evaluated the quantification of saffron extracts using similar methods [38,41]. This consistency supported the validity and dependability of our HPLC method for identifying and quantifying key saffron components, ensuring the purity and quality of the extract.

Plants abundant in phytochemicals are advantageous to health due to their potential to mitigate the risk of various conditions, including cardiovascular diseases, diabetes, and cancer [42]. The present study was conducted to evaluate the phytochemical constituents of saffron ethanolic extract (SEE). The findings revealed the presence of key bioactive compounds, particularly saponins and flavonoids, which were strongly detected. These compounds are known for their antioxidant, anti-inflammatory, and anticancer properties, which supports that SEE have significant therapeutic potential [43,44]. Tannins, which are polyphenolic compounds, exhibit a range of biological activities, including antimicrobial, anti-biofilm, and anticancer effects [45]. Meanwhile, steroids in medicinal plants are known for their diverse pharmacological effects, including cholesterol-lowering, anticarcinogenic, antibacterial, antifungal, and antidiabetic activities [46]. Tannins and steroids were present at moderate levels, which could contribute to antimicrobial, antioxidant, and anti-inflammatory activities found in SEE. The absence of alkaloids and terpenes suggests that these pathways are not prominent in saffron’s pharmacological profile when extracted with ethanol. SEE’s therapeutic effects are driven by the strong presence of saponins and flavonoids, with additional support from tannins and steroids [47]. The extract’s phytochemical profile highlights the importance of these compounds in saffron’s potential health benefits.

There are several mechanisms which contribute to the development of cataracts. One of the mechanisms is due to an increase in oxidative stress. Oxidative stress is a significant factor in the development of cataracts. Oxidative stress is the progressive and irreversible accumulation of oxidative damage caused by reactive oxygen species (ROS) which contributes to impaired physiological function and increased incidence of diseases [48], including cataracts. Some studies expressed that the mechanism of cataract development may occur through the diffusion of elevated lipid peroxidation products from the retina to the lens via the vitreous body in human cataractous lenses [8,48]. A case-control study of cataract patients revealed that they had greater levels of oxidative stress than healthy controls (Heydari et al., 2012). Previous research has reported that the mechanism of cataract development is due to iNOS expression and NF-kappa B activation. A previous study reported significant upregulation of iNOS mRNA and protein expression in Zucker diabetic fatty (ZDF) rat[49]. In the same study, the apoptotic change of lens epithelial cells was closely correlated with the expression pattern of iNOS, and accumulation of advanced glycation end products (AGEs) were observed in the cytoplasm [49]. Increased expressions of AGEs, NF-kappaB, and iNOS indicate the potential role of these factors in the apoptosis of LEC(lens epithelial cells) alterations that result in cataract formation [35].

Another mechanism that contributes to the development of cataracts is due to lipid changes in the blood. Lipids are an essential component of the human lens, and the degradation of membrane lipids significantly leads to cataractogenesis. Lipid synthesis is mainly responsible for growth and repair, and this could also be compromised in cataract lenses [50]. Research reported that serum lipid concentrations such as serum lipoprotein cholesterol (HDL-C), low-density lipoprotein cholesterol (LDL-C), triglyceride (TG), and cholesterol (CHO) are associated with cataracts [15]. Serum LDL-C and TG concentrations were found to be higher in the age-related cataract group compared to the control group, indicating that lowering the LDL-C and TG concentrations is associated with the progression of cataracts [15]. In our study, we found that feeding 1% HCD for 4 and 8 weeks could lead to the development of cataracts in rabbit’s lens. There is a significant increase of cataractous changes in HC4 and HC8 group compared to ND. HC4 and HC8 showed highest incidence of cataract changes (Grade 2), which indicates a strong link between hypercholesterolemia and cataract formation. In a previous study, it was reported that the mechanism of the development of cataracts in the retinas of NZWR fed with 2% HCD for 12 weeks could be due to the increased levels of H_2_O_2_ and a decrease in peroxidase activity [51]. Our findings are supported by past studies which reported cholesterol accumulation and increased immunoreactivity to glial fibrillary acidic protein (GFAP) in the retinas of cholesterol-fed rabbits when compared to control rabbits, indicating that high cholesterol diet could induce retinal degeneration and cataractous changes to rabbit lens [51].

In this study, NZWR rabbit lenses were used to observe the changes in the lens after saffron supplementation. The rabbit lens shares several key features with the human lens, particularly in terms of fiber arrangement and susceptibility to oxidative stress, making it a suitable model for studying cataractogenesis [52]. Numerous studies have used NZWR to investigate cataract and ocular conditions due to this similarity, and the findings are often used as preliminary data to guide human-based research [53,54]. The size and structure of the NZWR rabbit’s eye facilitate surgical procedures, as their ocular anatomy is similar to that of humans [55,56]. This similarity enhances the translational potential of findings from rabbit studies to human applications. The developmental timeline of the NZWR rabbit’s eye mirrors that of humans, providing a relevant model for understanding cataract pathology and treatment [57]. These findings demonstrated that rabbits are excellent subjects for use as models in ophthalmic research [58].

Saffron and its bioactive components (crocin, crocetin, safranal, and picocrocin) were found to exhibit anti-oxidant properties, anti-inflammatory and other therapeutic qualities [59]. Various bioactive properties of saffron were explored and discovered, highlighting its potential therapeutic applications [60]. The findings demonstrated that crocin and crocetin were responsible for its antioxidant activities [60,61]. In addition, crocin expressed its ability to scavenge free radicals and a wide range of pharmacological effects, such as hypolipidemic, antioxidant, anticancer, and antiatherosclerotic effects [61,62]. In our study, the rabbits were treated with 50 and 100 mg/kg/day saffron extracts and the findings showed that both doses of saffron extract could decrease the cataractous changes observed in the NZWR lens. Following post-treatment, there was also a reduction in the vacuolization and homogenization areas, postulating saffron’s protective effect against cataract progression. This is in line with a previous study which reported that 60 mg/kg saffron extract treatment for three weeks inhibited the development of selenite-induced cataracts in Wistar rats by suppressing the lipid peroxidation, protein oxidation, and proteolysis of lens proteins [63]. In another study by Bahmani et al. (2016) It was previously demonstrated that in the presence of high glucose concentrations, crocin(s) had an inhibitory effect on protein aggregation and AGEs formation in diabetic rats [64]. This indicates the ability of crocin to prevent proteins from protein glycation and aggregation which normally leads to cataract formation. This is aligned with past reports that saffron exhibits anti-inflammatory properties and plays a crucial role in the treatment of degenerative ocular disorders [29]. The findings revealed that saffron supplementation with a dose range of 15–50 mg/day resulted in substantial improvements in individuals with various ocular conditions [29]. We postulate that the therapeutic effect of saffron could be due to its free-radical scavenging activity [65]. Besides, the potential anti-cataractogenic potential of saffron could also be due to its antioxidant properties [63].

Despite strong evidence in our study, the results could not be translated into clinical settings yet. However, we used a valid animal model for this study. We recommend future research to investigate the mechanism of action of saffron in the pathway with a targeted focus on cataracts.

## Conclusion

Saffron treatment has demonstrated potential therapeutic properties with the amelioration of cataract lesions in atherosclerotic-induced New Zealand White Rabbits. These could be associated with the bioactive chemicals contained in saffron, including crocin, crocetin, and picrocrocin which are identified and verified through HPLC analysis. The findings of this study are consistent with previous research indicating that saffron has anti-inflammatory therapeutic effects. Extensive research is encouraged to investigate the underlying mechanism and effect of saffron in cataracts.

## Supporting information

S1 File HPLC data. (XLSX)
